# MakeSBML: a tool for converting between Antimony and SBML

**DOI:** 10.1515/jib-2024-0002

**Published:** 2024-06-11

**Authors:** Bartholomew E. Jardine, Lucian P. Smith, Herbert M. Sauro

**Affiliations:** Department of Bioengineering, University of Washington, Box 355061, Seattle, 98195, WA, USA

**Keywords:** systems biology, SBML, Antimony, software, modeling, reaction networks

## Abstract

We describe a web-based tool, MakeSBML (https://sys-bio.github.io/makesbml/), that provides an installation-free application for creating, editing, and searching the Biomodels repository for SBML-based models. MakeSBML is a client-based web application that translates models expressed in human-readable Antimony to the System Biology Markup Language (SBML) and vice-versa. Since MakeSBML is a web-based application it requires no installation on the user’s part. Currently, MakeSBML is hosted on a GitHub page where the client-based design makes it trivial to move to other hosts. This model for software deployment also reduces maintenance costs since an active server is not required. The SBML modeling language is often used in systems biology research to describe complex biochemical networks and makes reproducing models much easier. However, SBML is designed to be computer-readable, not human-readable. We therefore employ the human-readable Antimony language to make it easy to create and edit SBML models.

## 1 Introduction

To facilitate model sharing and reproducibility in the systems biology community the Systems Biology Markup Language (SBML) standard [1] is often used. However, this modeling standard can be difficult to read and edit without additional tools [2], [3], [4] and, in fact, was never designed for human consumption. Instead, various SBML editors have been developed [5, 6] to help users read and write SBML. In addition, at least two human-readable formats have been devised, SBML-shorthand (https://github.com/darrenjw/sbml-sh) and Antimony [7]. Antimony is a human-readable modeling language that greatly improves the ease of model creation and editing [7] of SBML-based models. A number of tools currently support Antimony, including Tellurium [2] and BioUML [6]. A software development kit, libAntimony (https://github.com/sys-bio/antimony), is provided in the form of a C/C++ library and associated optional Python bindings. The software kit provides a very simple API that allows the inter-conversion between Antimony and SBML.

In order to make the software development kit available to web browsers we have translated the C/C++ library into Web-assembly with associated JavaScript bindings. From this, we developed a client-side web application called MakeSBML (https://sys-bio.github.io/makesbml/) that allows a user to load, edit, and translate SBML and Antimony models from within the web browser. Because the application is a purely client-side application, MakeSBML is hosted by a GitHub page which makes the installation robust and relatively maintenance-free.

## 2 Materials and methods

### 2.1 Overview

The MakeSBML single-page web application uses a ported version of Antimony library [7] by translating the Antimony C++ library to JavaScript and WebAssembly https://webassembly.org/. This translation is done through Emscripten [8], a compiler toolchain used to compile existing C/C++ code and make it available for use through a web browser. The WebAssembly and JavaScript code (libantimonyjs) can be found at the GitHub site: https://github.com/sys-bio/libantimonyjs. This site includes detailed information on converting the Antimony library to JavaScript and WebAssembly. The MakeSBML website contains JavaScript calls to the Antimony library which passes back and forth, as a string, the Antimony or SBML model along with any error messages.

### 2.2 Usage

**Figure 1:**
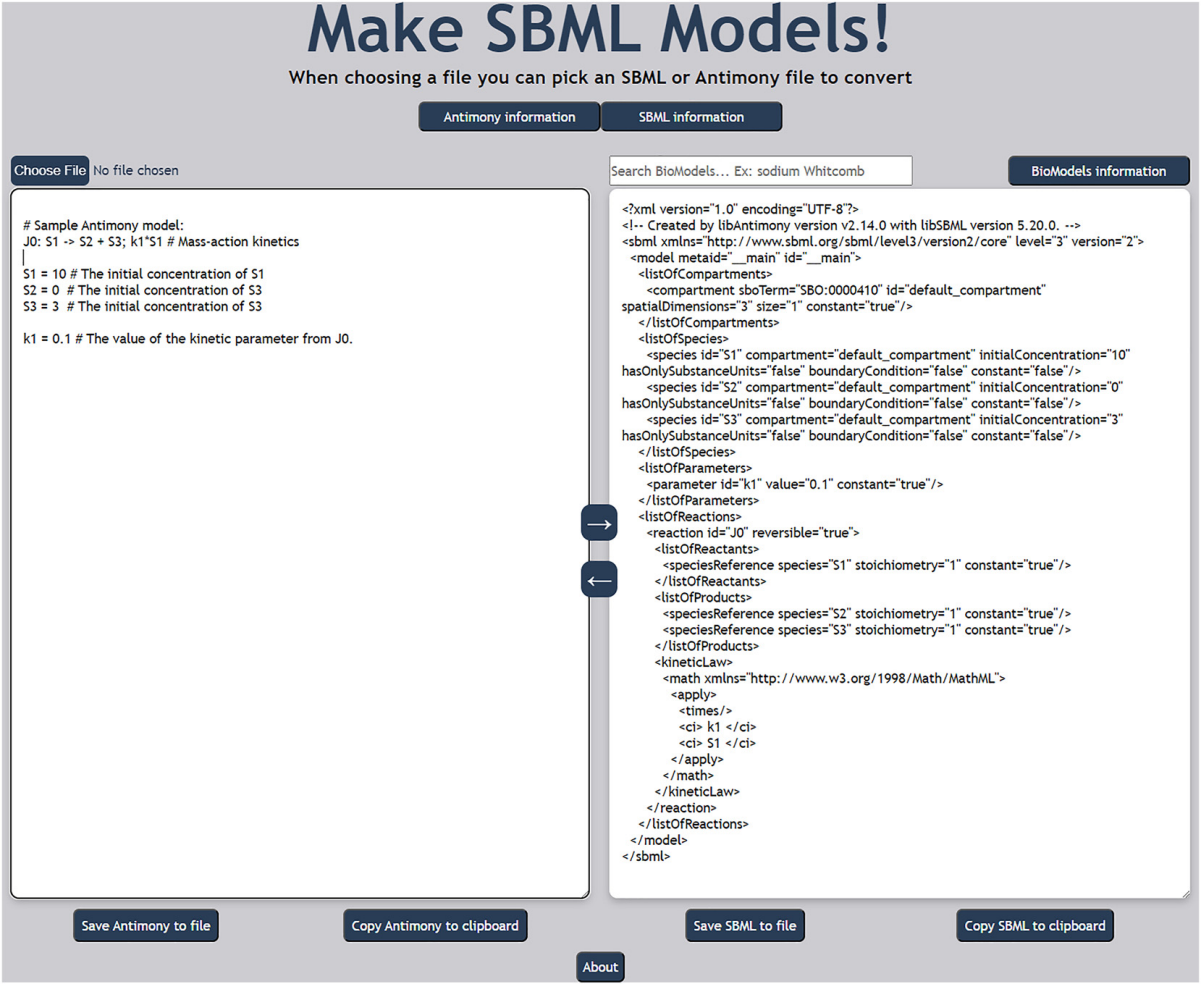
MakeSBML web application. Figure showing the Antimony model on the left, the translated SBML model on the right. The text search box above the translated SBML allows searching of BioModels model database (https://www.ebi.ac.uk/biomodels/) [9] for model download and translation to Antimony. Note the verbosity of the SBML model compared to the Antimony model.

Using the MakeSBML web application is straightforward. Go to https://sys-bio.github.io/makesbml/ and place either an Antimony or SBML model into the appropriate text box and press the arrow corresponding to the translation needed (Figure 1). A close inspection of a very simple, one reaction model in Figure 1 shows the verbosity needed in SBML (right panel), whereas Antimony (left panel) describes the reaction in simple text. When translating from Antimony to SBML, the Antimony library adds default values for any missing values required by the SBML specification (https://sbml.org/documents/specifications/). For instance note that a default compartment is added to the SBML model which was not explicitly declared in the Antimony model. Please see https://tellurium.readthedocs.io/en/latest/antimony.html for more information about using Antimony. Models can be loaded from the user’s computer, downloaded from the BioModels model database (https://www.ebi.ac.uk/biomodels/), pasted or typed directly into the appropriate text box. Model editing with MakeSBML makes it much simpler to create and edit SBML models. If your modeling software supports SBML but editing the model is difficult, editing with MakeSBML is often easier and quicker. Just load the SBML XML text in MakeSBML, edit the Antimony version, and save the translated SBML version for use in your simulation software of choice.

### 2.3 Implementation

The critical part of the MakeSBML implementation is the loading of the libAntimony JavaScript module as it calls a port of an existing library [7] to handle the Antimony – SBML translation. The use of an existing code library simplifies the MakeSBML implementation as the new code deals mainly with user interaction and not model translation. The loading of libAntimony is done through a call to the JavaScript wrapper libantimony.js which has mappings to the WebAssembly file libantimony.wasm. For MakeSBML, loading the libAntimony module in the browser looks like this:

libantimony().then((libantimony) => {

// Format: libantimony.cwrap(function name, return type, input param array of types).

loadString = libantimony.cwrap(“loadString”, “number”, [“number”]);

getSBMLString = libantimony.cwrap(“getSBMLString”, “string”, [“null”]);

getAntimonyString = libantimony.cwrap(“getAntimonyString”, “string”, [“null”]);

getLastError = libantimony.cwrap(“getLastError”, “string”, [“null”]);

jsAllocateUTF8 = (newStr) => libantimony.allocateUTF8(newStr);

});

*libantimony* is the name of the Antimony library and is accessed through the loading of the JavaScript wrapper contained in the file libantimony.js. The code then assigns a JavaScript variable, or alias, *loadString* to the Emscripten ’wrapped’ Antimony C function call, returns an integer and takes one number (pointer to a memory address containing the model string) as input. The other JavaScript aliases are *getSBMLString*, which returns the SBML version of the model, *getAntimonyString*, which returns the Antimony version, and *getLastError*, which returns any error found in the model. The final JavaScript alias *jsAllocateUTF8* is an Emscripten helper function *allocateUTF8()* that allocates memory for the string and creates a pointer to it. This function was added to the Antimony JavaScript wrapper when Empscripten compiled the original Antimony code.

Below is a JavaScript excerpt using the above JavaScript aliases for passing in an Antimony model and returning the translated model in SBML:

var ptrAntCode = jsAllocateUTF8(antCode);

var sbmlResult = “None”;

var load_int = loadString(ptrAntCode);

if (load_int > 0) {

sbmlResult = getSBMLString();

} else {

var errStr = getLastError();

}

To pass a JavaScript string into the Antimony function *loadString()* one must first call *jsAllocateUTF8()* to create a pointer to the Antimony model string. *loadString()* returns 0 if no errors are detected. After successfully loading the model the SBML can be retrieved by simply calling *getSBMLString()*. If an SBML model is loaded Antimony translation can be obtained with the *getAntimonyString()* function. See the GitHub repository https://github.com/sys-bio/makesbml for further details of MakeSBML implementation.

## 3 Discussion and conclusion

As modeling in systems biology becomes more complex, it is critical that researchers build upon existing work, as much time and money is invested in creating these mathematical models. The SBML standard for describing these models allows simple reproducibity of models between researchers since many simulation environments support SBML (https://en.wikipedia.org/wiki/List_of_systems_biology_modeling_software). To encourage reuse, the Antimony language provides clear text statements to describe these models allowing researchers an easier way to understand, edit, and update them for use in their own research, saving time and expense.

In this article, we describe MakeSBML, a client-side web tool for translating between SBML and Antimony. Because it uses only client-side technology (HTML, CSS, JavaScript, WebAssembly), MakeSBML can be hosted by servers that do not support server side processing that require languages such as Php, Python or Ruby. MakeSBML is currently on GitHub pages, which only supports static files. This makes it easy to port to other sites such as free resources like Google sites. Moreover, it reduces maintenance costs since an active server is not required and web technology tends to be more stable and backward compatible than equivalent desktop software. This allows tooling to continue to run long after the funding mechanism has ceased.

The use of Emscripten to generate WebAssemblies from existing C/C++ libraries and applications makes it easier for researchers to build, modify and simulate Systems Biology models from within their computer’s web browser. For educators, embedding interactive SBML models in their online learning materials will be invaluable, allowing the students to explore the behavior of chemical networks such as metabolic and signaling networks. Besides the Antimony library discussed here, the SBML library (libSBML) [10] has been converted to a JavaScript/WebAssembly version for use in a web browser (https://github.com/sys-bio/libsbmljs) [11]. This work includes a proof of concept simulator for existing SBML models (https://libsbmljs.github.io/).

MakeSBML provides a quick, and simple way to edit SBML models using the Antimony modeling language. By lowering the use barrier for researchers, MakeSBML takes the process a step further by encouraging model reuse and accelerates a user’s own model development and research as a whole.
